# Network analysis of the relationship between negative life events and depressive symptoms among adolescents: a comparison between males and females

**DOI:** 10.3389/fped.2025.1624250

**Published:** 2025-11-19

**Authors:** Ran Feng, Qiongzhi Zhang, Lingzhi Wang, Jingyu Lei, Xuerong Liu, Zhiyi Chen, Yanyan Li, Yujia Liao, Jie Gong, Jidong Ren, Xianyong An, Xuemei Li, Zuoshan Li

**Affiliations:** 1 Key Laboratory of Applied Psychology, Chongqing Normal University, Chongqing, China; 2 School of Psychology, Southwest University, Chongqing, China; 3 Experimental Research Center of Medical and Psychological Science (ERC-MPS), School of Psychology, Army Medical University, Chongqing, China; 4 Department of Clinical Psychology, Nanchong Psychosomatic Hospital (The Sixth People's Hospital of Nanchong), Nanchong, China; 5 School of Mechanical and Vehicle Engineering, Chongqing University, Chongqing, China

**Keywords:** adolescents, depression, negative life events, network analysis, gender difference

## Abstract

**Objective:**

To examine the network structure linking negative life events and depressive symptoms among adolescents in southwestern China, and to compare network characteristics between genders.

**Methods:**

Network analysis was used to assess associations between depressive symptoms and negative life events, identify core and bridge symptoms, evaluate global connectivity strength, and explore gender-specific differences in network structure. Participants completed the Center for Epidemiologic Studies Depression Scale (CES-D) and the Adolescent Self-Rating Life Events Checklist (ASLEC).

**Results:**

Data were collected from 104,552 adolescents aged 11–23 years. In the combined network, disease, academic stress, and being fined were the most central nodes, while flunking, broken heart, and academic stress served as key bridge symptoms between depressive symptoms and negative life events. Within the depressive symptom network, lack of happiness, depressed mood, feelings of failure, and sadness were the most central, with the strongest connection observed between hopelessness and feelings of failure. These core symptoms were consistent across genders. However, significant gender differences were found in network structure, global connectivity, and specific edges, with females exhibiting stronger overall symptom connectivity.

**Conclusions:**

The adolescent depression network is characterized by stable core symptoms and gender-specific differences in connectivity and bridge symptoms. These findings highlight the potential importance of bridge symptoms for early identification and for developing gender-sensitive intervention strategies.

## 1 Introduction

Adolescent depression is a major global public health concern and one of the leading causes of illness and disability among youth (1]). In China, depression has become one of the most prevalent and serious psychological disorders, posing significant risks to adolescents' emotional and social development (2).

Understanding adolescent depression remains challenging because of its heterogeneous symptom manifestations and multifactorial etiology (3). Traditional diagnostic models often assume the existence of an underlying disease entity that gives rise to observable symptoms. However, the Network Theory of Mental Disorders (NTMD) proposed by Borsboom (4) offers an alternative perspective, conceptualizing mental disorders as systems of directly interacting symptoms rather than as manifestations of a single latent cause. Within this framework, symptoms are represented as nodes, and their interconnections (e.g., co-activation or reinforcement) are depicted as edges in a network structure (5). Strong inter-symptom associations may create self-reinforcing feedback loops, rendering disorders persistent and resistant to spontaneous recovery. Importantly, NTMD suggests that interventions targeting central symptoms—those exerting strong influence within the network—can effectively disrupt these feedback loops and yield therapeutic benefits (4).

Aligned with this theoretical perspective, network analysis methods (NAM) have been widely adopted to investigate the structure of various psychopathological conditions, including depression. In such models, indices such as strength, closeness, and betweenness centrality are used to identify the most influential (“core”) symptoms, whereas bridge symptoms—nodes connecting distinct symptom communities—are identified as potential targets for intervention (6). Recent studies have applied network analysis to examine depressive symptoms patterns among adolescents (3, 7, 8). However, few of these studies have incorporated negative life events, a major category of environmental stressors that play a critical role in the onset and maintenance of depression.

The emergence of adolescent depression is shaped by multiple social and environmental factors, including family environment (9), school climate (10), and broader socioeconomic context (11). Empirical research has demonstrated that negative life events—such as interpersonal conflict, academic failure, family disruption, and social exclusion—are among the most salient external predictors of adolescent depression (12). Adolescents are particularly sensitive to such stressors, and cumulative exposure has been linked to increased severity and persistence of depressive symptoms (13). Within the framework of network theory, these stressors can be conceptualized as external activating nodes that interact with internal emotional and cognitive symptoms, forming tightly coupled systems that perpetuate psychological distress. Despite the conceptual importance of this interaction, few network-based studies have examined depressive symptoms and negative life events within a unified model. Furthermore, network structures are known to vary across cultural and regional contexts, and findings from one population cannot be assumed to generalize to another (56). This highlights the importance of conducting network analyses among underrepresented populations such as adolescents in southwestern China. Integrating depressive symptoms and negative life events within a single network may elucidate not only the core and bridge symptoms but also the dynamic pathways through which external stressors affect mental health outcomes.

Gender differences in depression have been well documented. Female adolescents typically exhibit higher levels of depressive symptoms than their male counterparts (14), and some studies indicate differences in the organization and connectivity of these symptoms. Although Mullarkey et al. (3) found no gender differences in global network strength, they reported significant structural variations in symptom connectivity. These findings raise an important question: does the interplay between negative life events and depressive symptoms differ by gender? Addressing this question could have critical implications for developing gender-sensitive intervention strategies.

Building upon the Network Theory of Mental Disorders, the present study aimed to: (a) construct a symptom network to identify the core symptoms of adolescent depression; (b) compare gender differences in the structure of depression networks; (c) incorporate negative life events into the network model to identify bridge symptoms that may serve as key pathways linking environmental stressors and depressive symptoms; and (d) examine gender differences in the integrated networks of depressive symptoms and negative life events. Based on previous findings, we proposed the following hypotheses: (H1) Core depressive symptoms would show the highest centrality; (H2) Significant gender differences would emerge in network structure and global strength; and (H3) Negative life events would function as bridge symptoms connecting external stressors and depressive symptoms, with potential gender-specific patterns.

## 2 Methods

### 2.1 Participants

Adolescents were recruited using cluster sampling, selecting entire classes from middle schools in Nanchong City, Sichuan Province, China. Only currently enrolled students were eligible to participate according to the inclusion criteria. To account for delayed school entry commonly observed in underdeveloped areas and to preserve data completeness, all valid responses were retained. As a result, the age range of participants extended beyond the typical adolescent span, which aligns with the extended definition of adolescence ranging from 10 to 24 years (15, 16). Demographic information, including gender, grade level, residential status, only-child status, left-behind status, parental education, and family relationship satisfaction, was collected for descriptive analysis.

### 2.2 Procedure

Data were collected online from middle school students in Nanchong City. Participation was voluntary, with anonymity and confidentiality ensured. Written informed consent was obtained from participants and their legal guardians, as well as approval from the participating schools. The study strictly adheres to the ethical principles outlined in the 1975 Declaration of Helsinki and has received approval from the Ethics Committee of Nanchong Psychosomatic Hospital (Approval No. NCPP 2022002).

### 2.3 Measurement

Depressive symptoms were assessed using the Chinese version of the Center for Epidemiological Studies Depression Scale (CES-D) (17). The CES-D includes 20 items evaluating depressive symptomatology on a 4-point Likert scale (0–3), with items 4, 8, 12, and 16 reverse scored. The CES-D has demonstrated strong psychometric properties in Chinese adolescent populations (18–20). In the present study, the Cronbach's *α* coefficient was 0.936.

Negative life events were measured using the Adolescent Self-Rating Life Events Checklist (ASLEC) (21), which includes 27 items covering six domains: interpersonal relationships, academic stress, punishment, loss, health adaptation, and other events. The ASLEC uses a 5-point scale, with total scores ranging from 27 to 135. Higher scores indicate greater cumulative stress. The ASLEC has been widely used among Chinese adolescents and demonstrates good reliability (22). In this study, the Cronbach's *α* was 0.938.

### 2.4 Statistical analysis

Three main objectives guided the statistical analyses: (a) identifying core depressive symptoms (H1); (b) assessing gender differences in network structure and global connectivity (H2); (c) integrating negative life events to detect bridge symptoms between environmental stressors and depression (H3).

All statistical analyses were conducted using R (version 4.4.1).

Descriptive statistics and reliability analyses were performed using the R package psych. For the CES-D, we examined the mean, standard deviation, skewness, and kurtosis of all items. Because response distributions were skewed, items were dichotomized such that “0” indicated absence of a symptom and “1–2” indicated presence, with positively worded items reverse-coded. Although dichotomization can reduce information and statistical power (23), this approach minimizes distortions from non-normality, is commonly employed in large-scale network research, and facilitates comparability with prior studies of adolescent depression (24).

Network estimation and visualization were performed in R using the *qgraph* package, following established procedures for psychological network analysis (25, 26). The Ising model is particularly suited for binary data and allows the estimation of conditional dependencies among symptoms while controlling for all other nodes, providing a more accurate representation of the direct associations between symptoms and life events (27). In the estimated networks, nodes represented CES-D or ASLEC items, and edges reflected conditional associations between items after accounting for all other variables.

Core symptoms were identified through centrality analyses. Three centrality indices were calculated: strength centrality, defined as the sum of the absolute edge weights of a node; closeness centrality, the inverse of the average shortest path length between a node and all other nodes; and betweenness centrality, which quantifies how frequently a node lies on the shortest path between two other nodes. Among these, strength centrality is generally considered the most robust indicator in psychological networks. In addition, bridge strength centrality was calculated to detect symptoms that connect different symptom communities, specifically depressive symptoms and negative life events. Identifying bridge nodes is crucial for clarifying cross-domain activation and for revealing clinically meaningful targets that may mediate the impact of environmental stressors on depression (28, 57). This approach has also been applied in recent network studies outside the depression domain, such as Yang et al. (19, 20), who demonstrated the utility of bridge analysis in linking physical exercise behaviors with mental health symptoms in adolescents.

To assess gender differences (H2, H3), networks were estimated separately for females and males. Network Comparison Tests (NCT) were performed using the NCT package (29), with three permutation-based procedures: (a) a network structure invariance test, assessing whether the global pattern of edges differed between groups; (b) a global strength invariance test, comparing overall connectivity; and (c) a centrality invariance test, evaluating the stability of central nodes across genders. A total of 5,000 permutations were used to ensure robustness. Edge-specific differences were further examined, with Holm–Bonferroni correction applied to adjust for multiple comparisons.

## 3 Results

### 3.1 Descriptive statistics

A total of 104,552 adolescents participated in the study, including 53,704 females (51.4%) and 50,848 males (48.6%), aged 9–23 years (*M* = 14.51, SD = 1.76). The grade distribution was as follows: 7th (14.3%), 8th (21.2%), 9th (20.5%), 10th (11.1%), 11th (16.5%), and 12th (16.4%). Demographic variables, including gender, grade level, residential status, only-child status, left-behind status, parental education, and family relationship satisfaction, are summarized in Table 1.

**Table 1:** Demographic characteristics (*n* = 104,552).

Demographic	*M* (SD) or *N* (%)
Age	14.51 (1.76)
Gender	
Male	50,848 (48.6%)
Female	53,704 (51.4%)
Grade	
7th Grade	14,906 (14.3%)
8th Grade	22,175 (21.2%)
9th Grade	21,484 (20.5%)
10th Grade	11,570 (11.1%)
11th Grade	17,269 (16.5%)
12th Grade	17,148 (16.4%)
Living dormitory	
Yes	45,360 (43.4%)
No	59,192 (56.6%)
Only child	
Yes	18,360 (17.6%)
No	86,192 (82.4%)
Residence	
Urban	49,094 (47.0%)
Town	31,193 (29.8%)
Rural	24,265 (23.2%)
Left-behind experience	
Yes	52,980 (50.7%)
No	51,572 (49.3%)
Parental educational attainment	
Secondary school or below	62,966 (60.2%)
University degree	8,580 (2.8%)
Postgraduate degree	208 (0.2%)
Unknown	32,798 (31.4%)
Family relationship satisfaction	
Very satisfied	38,221 (36.6%)
Somewhat satisfied	38,064 (36.4%)
Neutral	21,915 (20.9%)
Somewhat dissatisfied	4,671 (4.5%)
Very dissatisfied	1,681(1.6%)

The prevalence of depression was 24.9%, with 26,033 individuals scoring above the threshold, and the high-risk group consisted of 8,320 individuals (8.0%). The mean, standard deviation, skewness, kurtosis, and frequency of the CES-D symptoms are reported in Table 2. The overall mean and standard deviation of all symptoms are *M* = 0.54, SD = 0.52. The symptoms of difficulty with concentrating (*M* = 0.90, SD = 0.87) and feeling bothered (*M* = 0.75, SD = 0.82) had the highest mean ratings, indicating higher frequency and intensity of these symptoms among participants. Conversely, the symptoms of inability to get going (*M* = 0.19, SD = 0.53) and people unfriendly (*M* = 0.36, SD = 0.68) had the lowest mean ratings, suggesting lower prevalence of these symptoms. The sample's diversity in terms of gender, grade level, only child status, and residence is representative of the population from which the participants were drawn.

**Table 2:** Mean, standard deviation, skewness, and kurtosis, and frequency of the CES-D symptoms (*n* = 104,552).

Symptoms	CES-D	Mean	SD	Skewness	Kurtosis	%Absence (“0”)	%Presence (“1”)
Feeling bothered	1	0.75	0.82	1.01	0.52	45.05	54.95
Appetite changes	2	0.45	0.69	1.62	2.48	64.70	35.30
Feeling blue	3	0.49	0.77	1.61	2.10	64.00	36.00
Lack of feeling good	4	0.74	0.89	1.08	0.35	49.54	50.46
Difficulty with concentrating	5	0.90	0.87	0.79	−0.01	36.68	63.32
Depressed mood	6	0.70	0.82	1.06	0.54	48.68	51.32
Everything was an effort	7	0.63	0.79	1.18	0.95	52.27	47.73
Hopelessness	8	0.61	0.85	1.37	1.13	58.04	41.96
Feeling of failure	9	0.48	0.79	1.71	2.31	66.37	33.63
Fearful	10	0.49	0.75	1.61	2.22	63.43	36.57
Sleep disturbances	11	0.58	0.83	1.42	1.30	59.26	40.74
Lack of happiness	12	0.60	0.78	1.27	1.21	54.64	45.36
Talking less	13	0.42	0.72	1.84	3.07	68.44	31.56
Lonely	14	0.50	0.79	1.65	2.12	64.76	35.24
People unfriendly	15	0.36	0.68	2.11	4.27	73.68	26.32
Lack of enjoyment	16	0.37	0.72	2.12	4.07	74.14	25.86
Crying	17	0.59	0.78	1.36	1.50	55.43	44.57
Sadness	18	0.59	0.79	1.36	1.36	56.73	43.27
Feeling disliked by others	19	0.39	0.72	1.99	3.61	71.60	28.40
Inability to get going	20	0.19	0.53	3.30	11.69	86.05	13.95

### 3.2 Depressive symptoms network analysis

The CES-D item measuring the tendency of engagement into inability to get going showed a skewed distribution (skewness = 3.30), with 86.05% of participants reporting the absence of this symptom (Table 2). Moreover, when compared to the mean level of informativeness of the CES-D items (0.26 ± 0.71), item 20 was not within 2.5 standard deviations below the mean, indicating it still provided sufficient information. For these reasons, despite its high skewness, item 20 was retained in the subsequent analysis. No items were excluded based on their informativeness or distribution characteristics, resulting in the inclusion of all 20 items in the subsequent analyses. In line with H1, we first constructed the depressive symptom network to identify core symptoms.

**Figure 1:**
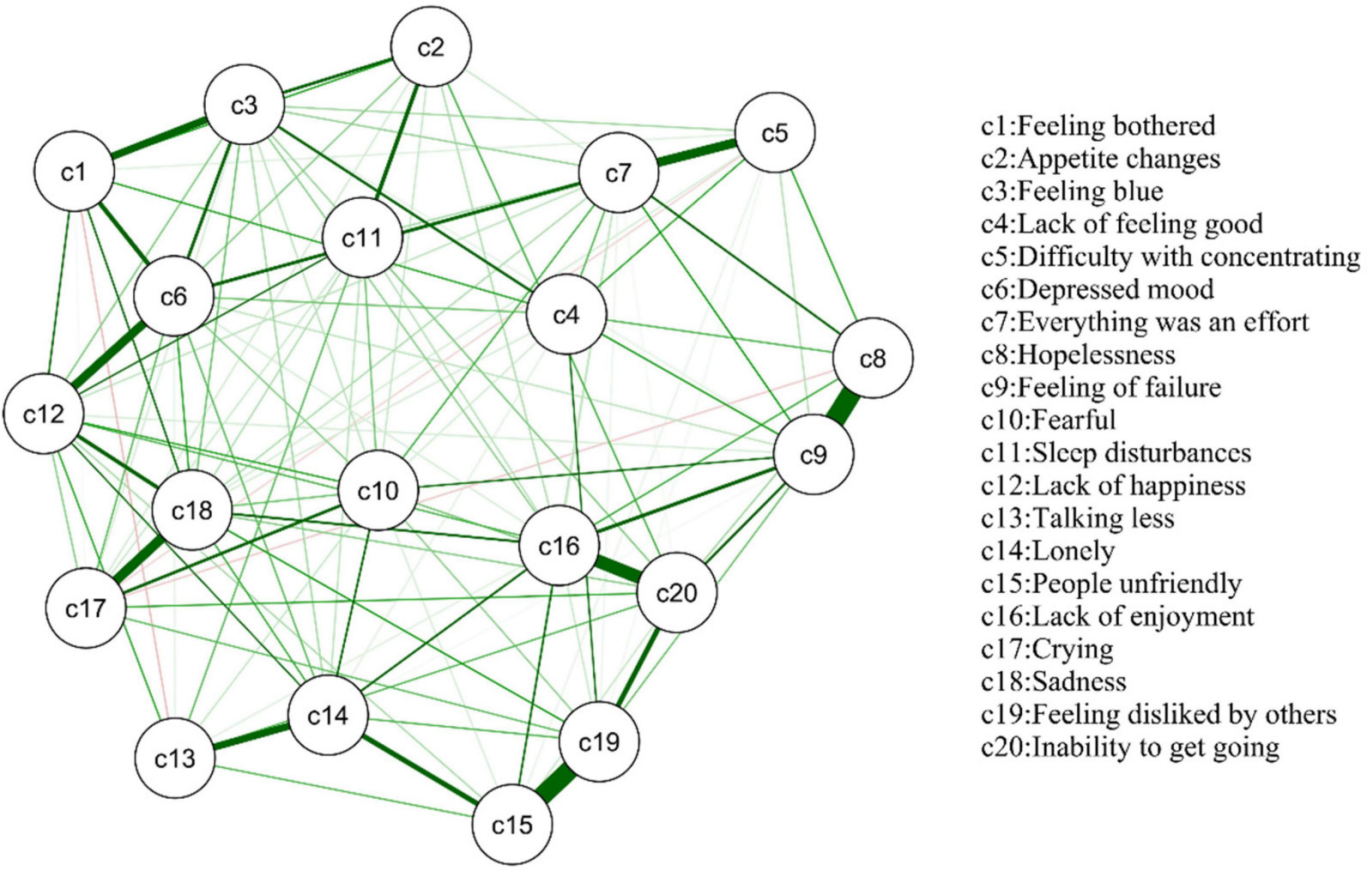
GLASSO network model for depressive symptoms in the total sample (*n* = 104,522). The thickness of the edges indicates the strength of the association, with thicker edges representing stronger connections (green = positive, red = negative).

**Figure 2:**
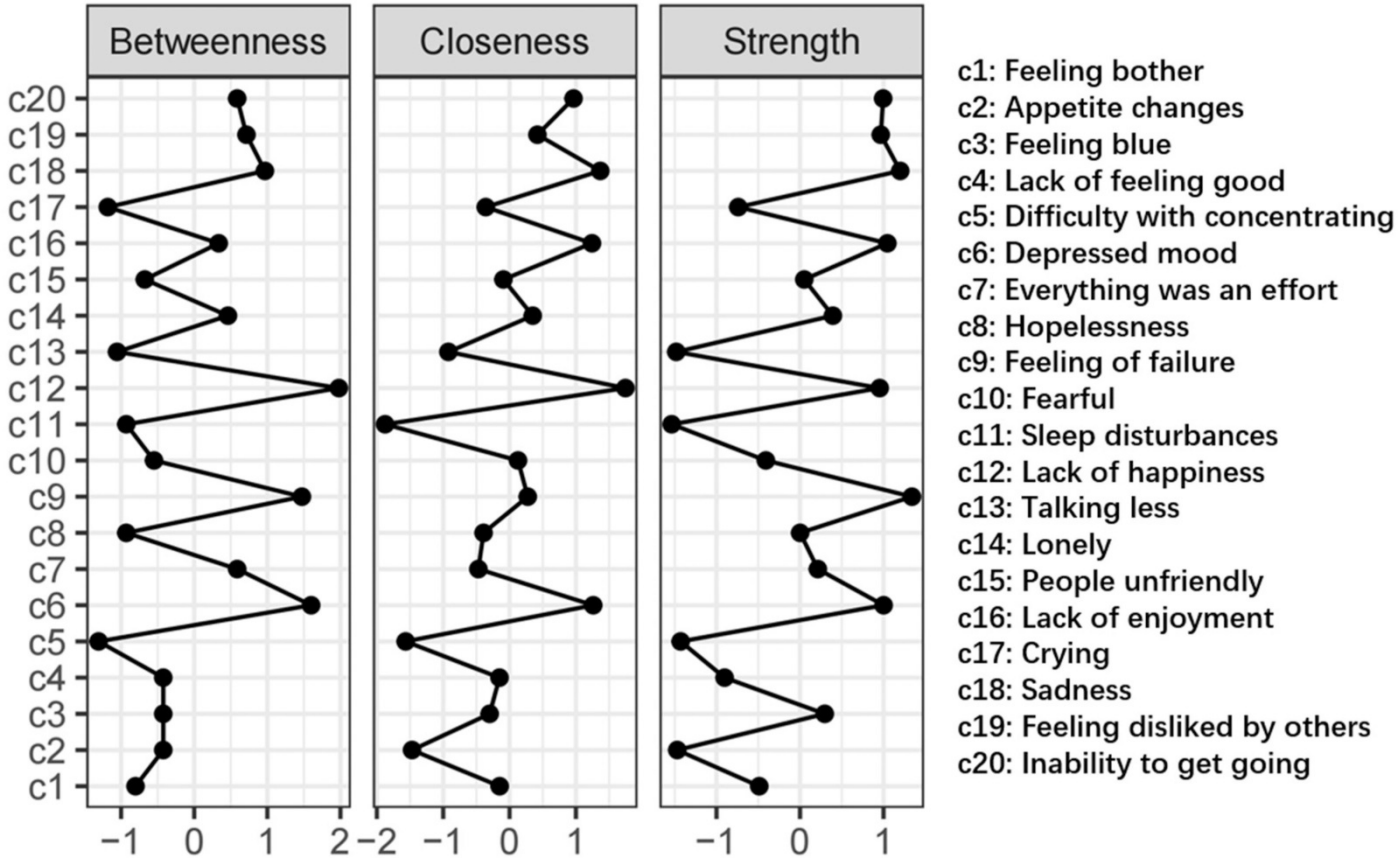
Centrality indices of depressive symptoms, shown as standardized values *z*-scores. The *x*-axes represent standardized centrality values, with higher values indicating greater centrality.

The network of depressive symptoms, as estimated with the Ising model, is shown in Figure 1, while Figure 2 shows the network's centrality indexes, namely strength, betweenness and closeness. Table 3 shows all adolescents the most central symptoms in the network are lack of happiness (Bet = 26, Clo = 0.00397, Str = 1.124), depressed mood (Bet = 23, Clo = 0.00380, Str = 1.134), feeling of failure (Bet = 22, Clo = 0.00345, Str = 1.206) and sadness (Bet = 18, Clo = 0.00384, Str = 1.176). In the sample, the strongest connection was observed between hopelessness and feeling of failure. Additionally, strong connections were found between people unfriendly and feeling disliked by others, lack of enjoyment and inability to get going, difficulty with concentrating and everything was an effort, crying and sadness. These connections indicate a high level of association among these symptom pairs within the network. In summary, symptoms such as lack of happiness, depressed mood, feeling of failure, and sadness emerged as the most central, supporting H1.

**Table 3:** Centrality of depressive symptoms within the adolescent population (overall) and among male and female adolescents.

Symptoms	Adolescents (overall)			Male			Female		
	Bet	Clo	Str	Bet	Clo	Str	Bet	Clo	Str
1	4	0.00330	0.818	2	0.00316	0.732	5	0.00386	0.802
2	7	0.00283	0.610	8	0.00299	0.575	3	0.00273	0.616
3	7	0.00324	0.984	7	0.00329	0.981	7	0.00322	0.967
4	7	0.00330	0.730	8	0.00335	0.731	7	0.00330	0.714
5	0	0.00279	0.619	0	0.00277	0.595	1	0.00288	0.620
6	23	0.00380	1.134	29	0.00396	1.116	28	0.00386	1.140
7	15	0.00318	0.967	19	0.00323	0.945	15	0.00325	0.982
8	3	0.00321	0.922	0	0.00318	0.926	3	0.00325	0.907
9	22	0.00345	1.206	20	0.00344	1.204	23	0.00349	1.215
10	6	0.00340	0.835	2	0.00334	0.821	7	0.00345	0.821
11	3	0.00268	0.596	1	0.00269	0.573	5	0.00272	0.621
12	26	0.00397	1.124	20	0.00397	1.113	26	0.00398	1.111
13	2	0.00302	0.607	0	0.00305	0.554	4	0.00296	0.616
14	14	0.00348	1.005	16	0.00377	1.035	13	0.00339	0.998
15	5	0.00332	0.932	5	0.00339	0.950	9	0.00334	0.932
16	13	0.00380	1.143	29	0.00392	1.168	7	0.00373	1.118
17	1	0.00322	0.765	1	0.00340	0.657	0	0.00321	0.785
18	18	0.00384	1.176	21	0.00404	1.149	13	0.00359	1.179
19	16	0.00350	1.127	13	0.00357	1.110	15	0.00347	1.123
20	15	0.00370	1.133	19	0.00378	1.147	15	0.00374	1.134

### 3.3 Network comparisons between male and female

**Figure 3:**
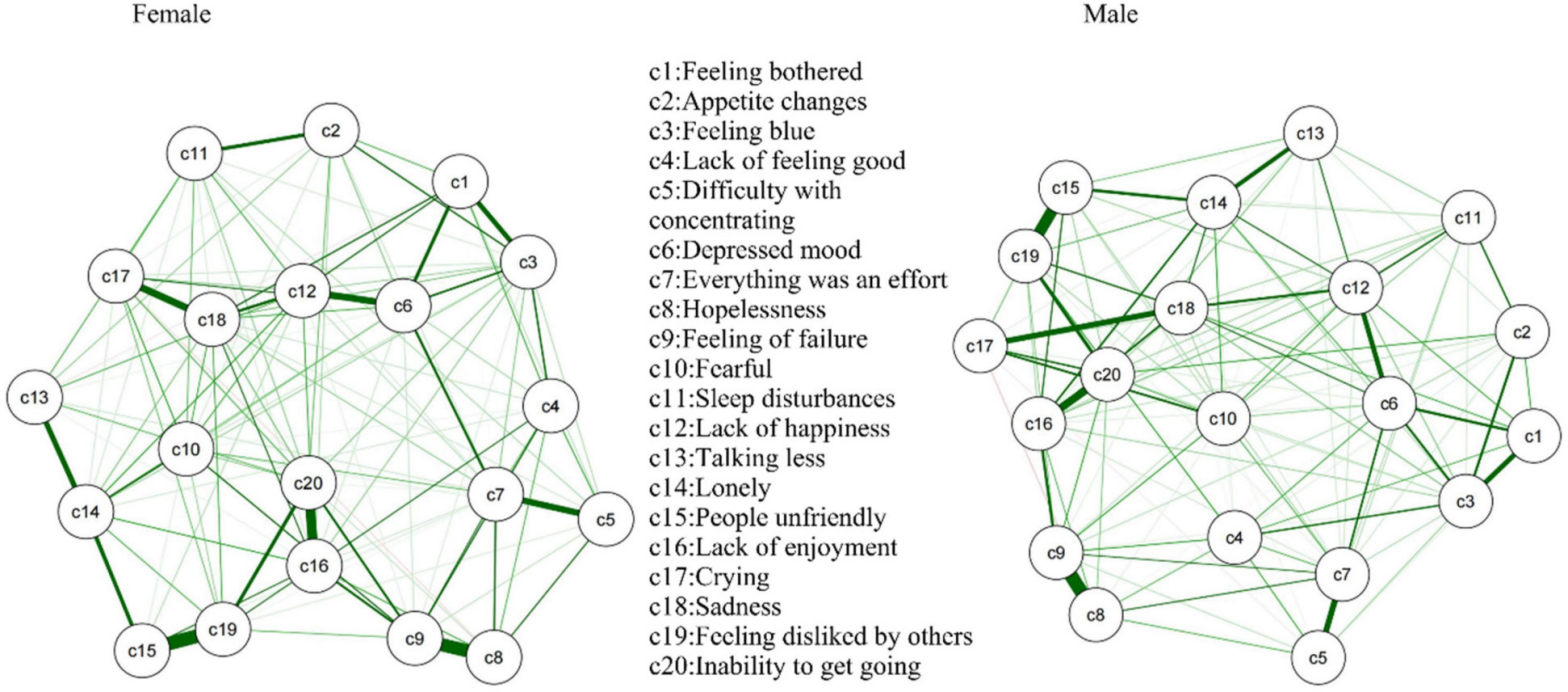
GLASSO network model for depressive symptoms in males (*n* = 50,848) and females (*n* = 53,704). The thickness of the edges indicates the strength of the association, with thicker edges representing stronger connections (green = positive, red = negative).

Figure 3 shows the network built on the depressive symptoms across males (*n* = 50,848) and females (*n* = 53,704). Table 3 shows the most central symptoms in male network are depressed mood (Bet = 29, Clo = 0.00396, Str = 1.116), lack of enjoyment (Bet = 29, Clo = 0.00392, Str = 1.168), sadness (Bet = 21, Clo = 0.00404, Str = 1.149), feeling of failure (Bet = 20, Clo = 0.00344, Str = 1.204) and lack of happiness (Bet = 20, Clo = 0.00397, Str = 1.113). The most central symptoms in female network are depressed mood (Bet = 28, Clo = 0.00386, Str = 1.140), lack of happiness (Bet = 36, Clo = 0.00398, Str = 1.111) and feeling of failure (Bet = 23, Clo = 0.00349, Str = 1.215). Despite the lack of significant differences in certain specific edges, the overall network structure showed a significant difference (*p* < 0.001). To address H2, we compared network structures between male and female adolescents.

#### 3.3.1 Network invariance test and global strength invariance

The network invariance test yielded a test statistic of *M* = 0.058, with a *p*-value of 0.001, indicating a significant difference in the overall structure of the depression symptom networks between genders. The global strength for males was 9.09, while for females, it was 9.30, with a test statistic of S = 0.210 and a *p*-value of 0.001. Although the difference in global strength is relatively small, it is statistically significant, indicating that the overall connectivity among depressive symptoms varies between genders.

#### 3.3.2 Edge invariance test

Several edges displayed significant differences in connectivity strength. For instance, symptom pairs c1–c2 (*p* < 0.05), c2–c4 (*p* < 0.001), and c4–c6 (*p* < 0.001) demonstrated significant variations between genders. These results indicate that certain symptom pairs exhibit differential associations between males and females, warranting further examination of gender-specific interaction patterns within these pairs.

#### 3.3.3 Centrality invariance test

Although there were no significant differences in the core symptom nodes, some nodes demonstrated significant differences in centrality, including c1, c2, c3, c7, and c10 (*p* < 0.01). In particular, nodes c1 and c17 showed marked differences in both strength and expected influence, indicating that these symptoms occupy distinct central roles in the male and female networks.

### 3.4 ASLEC-CES-D network analysis

**Figure 4:**
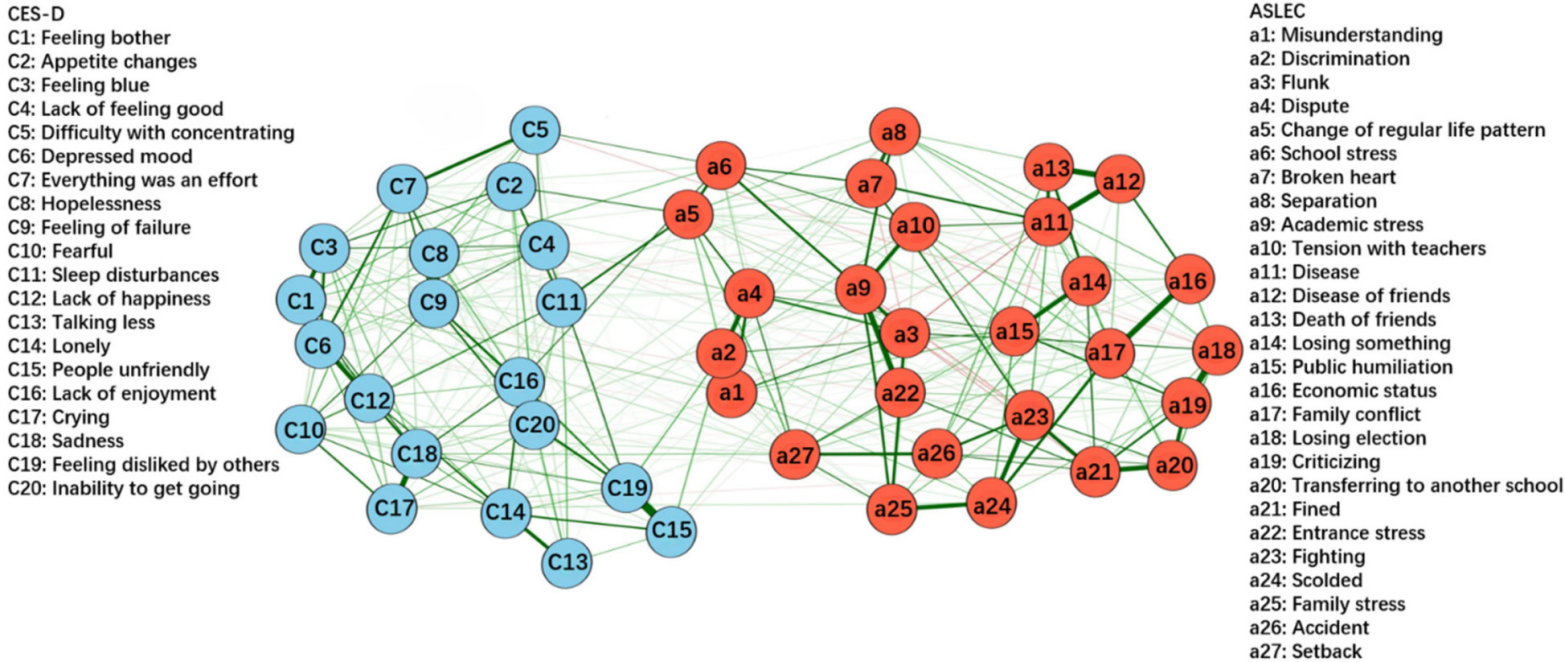
GLASSO network model for depressive symptoms and negative life events in total sample (*n* = 104,522). The thickness of the edges indicates the strength of the association, with thicker edges representing stronger connections (green = positive, red = negative).

**Figure 5:**
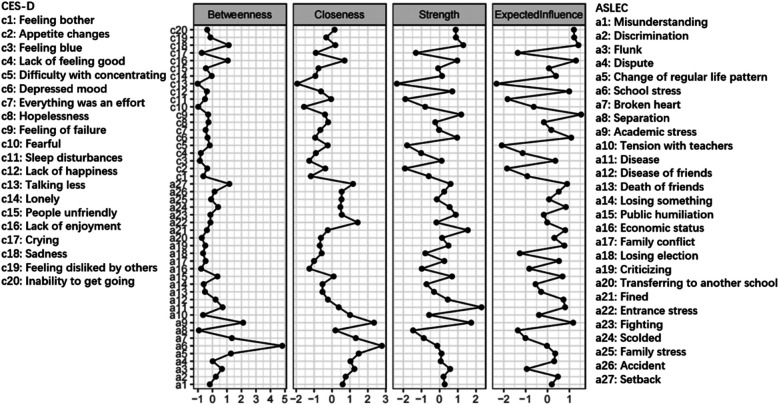
Centrality indices of depressive symptoms and negative life events in total sample (*n* = 104,522). The *x*-axes represent standardized centrality values, with higher values indicating greater centrality.

Figure 4 shows the network constructed based on depressive symptoms and negative life events in the total sample (*n* = 104,552). Figure 5 illustrates that the most central symptoms in this network are disease (Bet = 78, Clo = 0.00097, Str = 1.411), academic stress (Bet = 107, Clo = 0.00117, Str = 1.307), and fined (Bet = 142, Clo = 0.00091, Str = 1.273). Several bridge symptoms were identified between the depressive symptom and negative life event communities. The symptoms with the highest bridge strength centrality were flunk (BS = 0.70), broken heart (BS = 0.49) and academic stress (BS = 0.47). In relation to H3, we incorporated negative life events into the network to identify bridge symptoms.

### 3.5 ASLEC-CES-D network comparisons between male and female

Figuress 6, 7 respectively present the networks constructed based on depressive symptoms and negative life events in females (*n* = 53,704) and males (*n* = 50,848). Figure 8 illustrates that the most central symptoms in female network are academic stress (Bet = 169, Clo = 0.00119, Str = 1.39), disease of friends (Bet = 77, Clo = 0.00099, Str = 1.34), and sadness (Bet = 79, Clo = 0.00095, Str = 1.25). Figure 9 illustrates that the most central symptoms in male network are disease (Bet = 99, Clo = 0.00099, Str = 1.491), Lack of enjoyment (Bet = 101, Clo = 0.00099, Str = 1.192), and sadness (Bet = 97, Clo = 0.00096, Str = 1.191). Several bridge symptoms were identified between the depressive symptom and negative life event communities. In the female network, the symptoms with the highest bridge strength centrality were change of regular life pattern (BS = 0.33), school stress (BS = 0.32) and setback (BS = 0.18). In the male network, the symptoms with the highest bridge strength centrality were school stress (BS = 0.26), change of regular life pattern (BS = 0.25), and setback (BS = 0.15). Although not all individual edges demonstrated statistically significant differences, the overall network structure differed significantly between the female and male groups (*M* = 0.071, *p* < 0.001). Moreover, global strength—representing the total level of connectivity within the network—was significantly higher in the female network compared to the male network (S = 0.526, *p* < 0.05). Notably, several specific edges, such as those between discrimination and flunk or academic stress and disease, exhibited significant group differences. Extending H3, we further examined gender differences in the integrated ASLEC-CES-D network.

**Figure 6:**
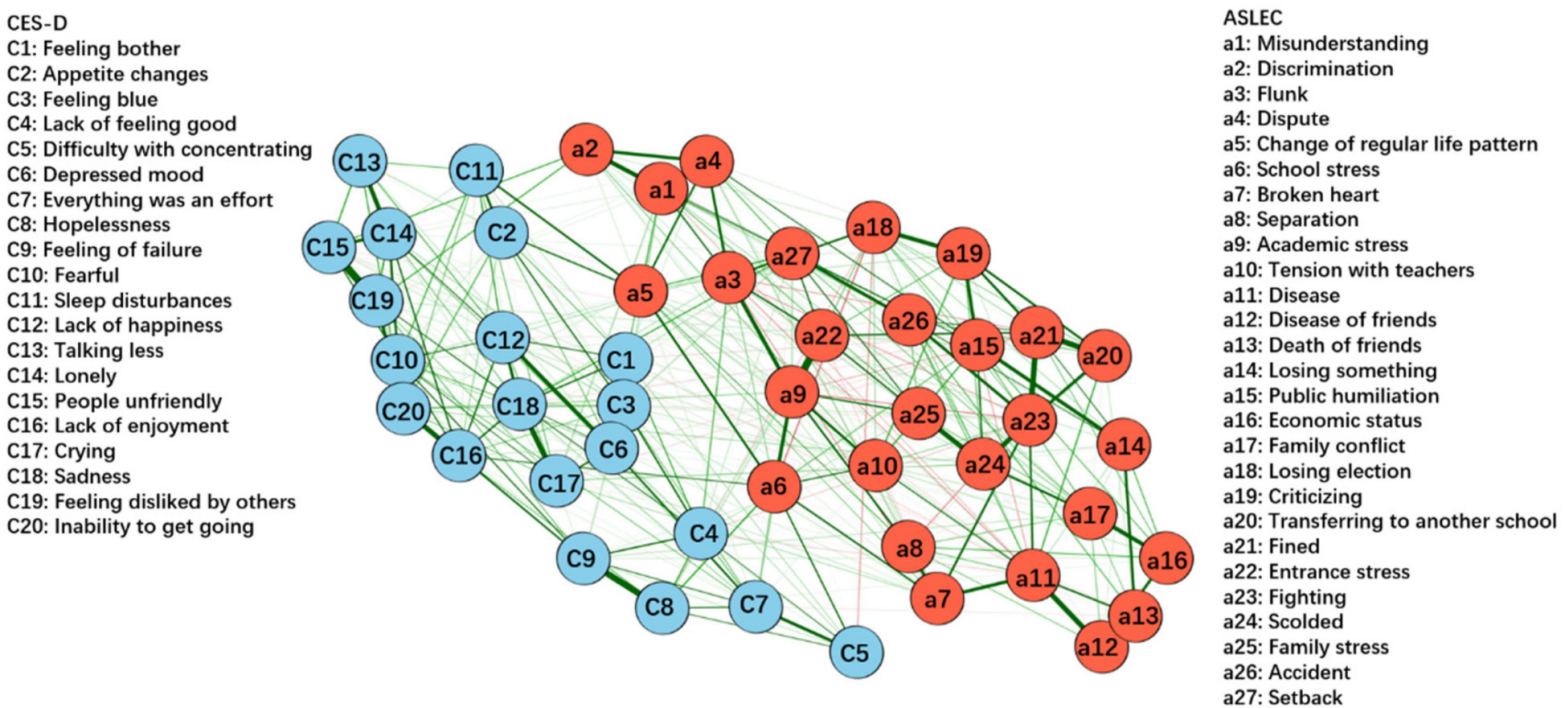
GLASSO network model for depressive symptoms and negative life events in females (*n* = 53,704). The thickness of the edges indicates the strength of the association, with thicker edges representing stronger connections (green = positive, red = negative).

**Figure 7:**
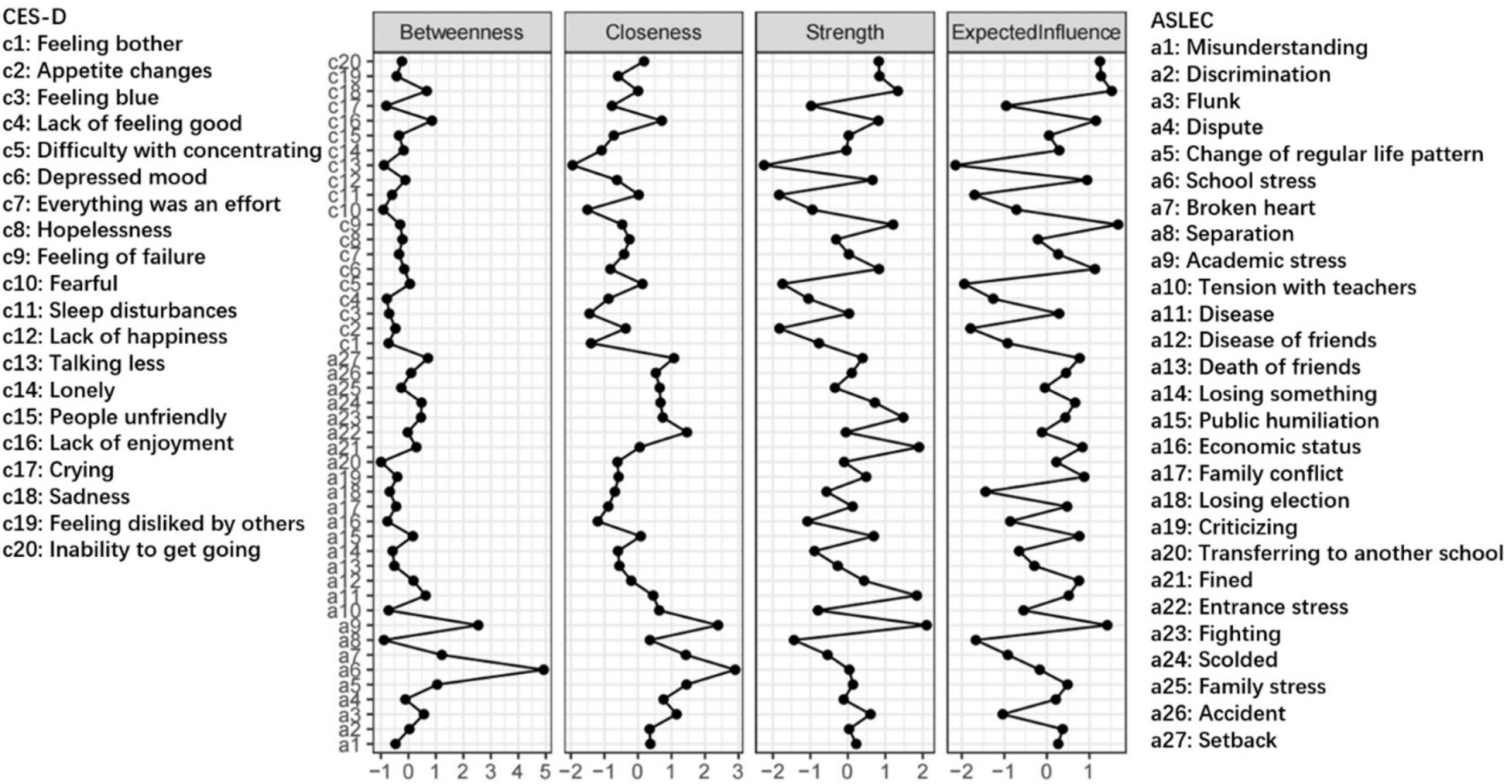
GLASSO network model for depressive symptoms and negative life events in males (*n* = 50,848). The thickness of the edges indicates the strength of the association, with thicker edges representing stronger connections (green = positive, red = negative).

**Figure 8:**
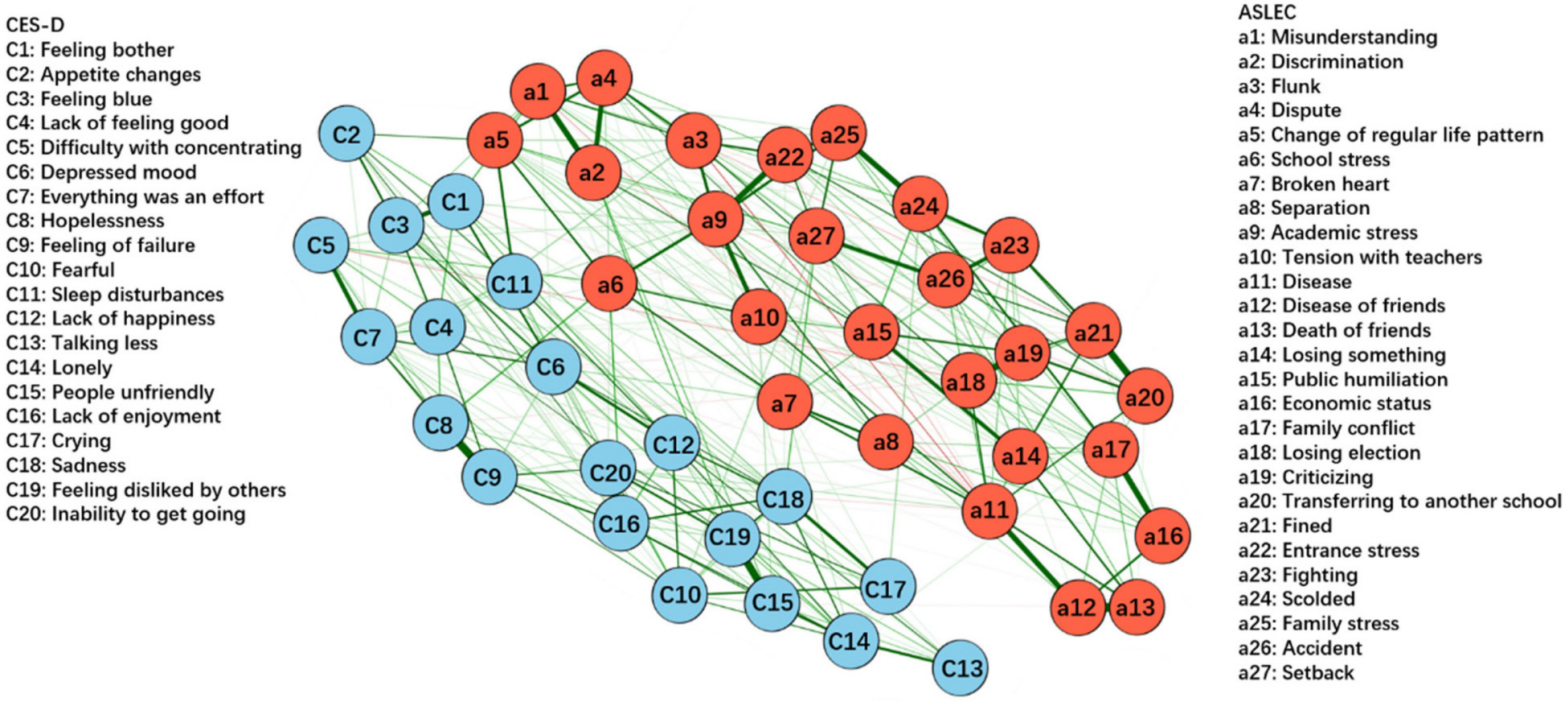
Centrality indices of depressive symptoms and negative life events in females (*n* = 53,704). The *x*-axes represent standardized centrality values, with higher values indicating greater centrality.

**Figure 9:**
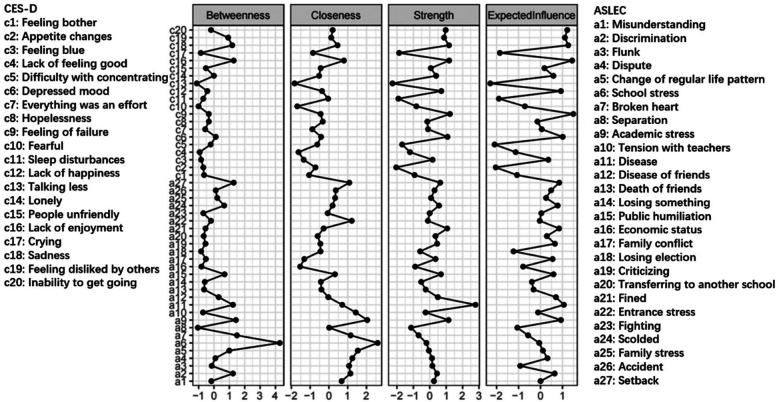
Centrality indices of depressive symptoms and negative life events in males (*n* = 50,848). The *x*-axes represent standardized centrality values, with higher values indicating greater centrality.

## 4 Discussion

Using data from 104,552 adolescents in southwestern China, this study mapped depressive symptom networks and their associations with negative life events, identifying core and bridge symptoms as well as significant gender-specific differences in network structure and connectivity. These findings provide valuable implications for targeted prevention and intervention strategies.

Consistent with previous evidence, the core symptoms of depression among adolescents in southwestern China were lack of happiness, depressed mood, feeling of failure and sadness. As noted by Beard et al. (30), sadness or depressed mood is a hallmark symptom for diagnosing Major Depressive Disorder according to the DSM. Lack of happiness and lack of enjoyment are also core depressive symptoms, aligning with findings from other populations (31, 32). Similar to Indian adolescents (33), feeling of failure also emerged as a central symptom among Chinese adolescents.

In the Chinese sociocultural context, strong emphasis is placed on academic achievement and social status (34). Success is often defined by academic performance, which creates immense pressure on adolescents to excel. Failure to meet high expectations from teachers, parents, and themselves can lead to profound feelings of inadequacy and disappointment, fostering a deep sense of failure (35). Academic pressure—defined as a student's perception of academic demands that exceed adaptive capacity—is one of the most common stressors among adolescents, particularly in China, where education is highly valued (36). The fear of academic failure may lead to chronic stress and anxiety, which are key contributors to depressive symptoms (37). This may also explain why the strongest connection in the network was observed between hopelessness and feeling of failure.

In the overall network combining depressive symptoms and negative life events, disease, academic stress, and being fined emerged as the most central nodes. Consistent with longitudinal findings, adolescents with chronic physical health conditions tend to exhibit higher levels of depressive symptoms throughout adolescence (38). Physical illness not only causes direct physiological discomfort but can also trigger a series of adverse psychosocial consequences, such as social isolation (39), academic disruption (40), and decreased self-worth (41). These negative experiences heighten perceived stress and negative emotions, thereby triggering or exacerbating depressive symptoms (42). Such mechanisms may help explain the particularly high centrality of the disease node in the integrated network.

In the context of traditional Chinese culture, respect for authority and obedience are regarded as core values, and corporal punishment and strict disciplinary measures remain prevalent in both families and schools. Research has shown that being fined or punished not only causes physical harm but also induces feelings of shame among adolescents (43). Prolonged exposure to such environments may lead adolescents to suppress their emotions (13), thereby increasing their vulnerability to depressive symptoms (44).

This study also identified bridge symptoms within the ASLEC-CES-D network. Bridge symptoms are conceptualized as transmission pathways through which one disorder may spread to another. Therefore, when a disorder emerges, intervening on potential bridge symptoms can effectively prevent symptom spillover and the development of comorbid conditions (45). In the present study, flunk, broken heart, and academic stress function as bridge nodes between negative life events and depressive symptoms, potentially facilitating cross-network links from external stressors to internalizing emotional problems. These nodes are not only independent risk factors but may also amplify the overall impact of stress by activating core depressive symptoms. Academic stress and academic failure, in particular, hold cultural significance among Chinese adolescents, where academic performance is strongly tied to self-worth and family honor. The broken heart node, meanwhile, reflects developmental challenges in peer and intimate relationships during adolescence; such relational setbacks are closely linked to depressive symptoms (46, 47). Targeting bridge symptoms—especially those with high bridge centrality—through tailored interventions may thus help reduce the risk of depression and comorbid distress.

Using network analysis, the present study identified significant gender differences in the CESD and ASLEC-CESD combined networks in terms of network structure, global connectivity strength, edge invariance, and centrality invariance. The female network exhibited a more densely connected structure and significantly higher global connectivity strength compared to the male network, which may partially explain the higher prevalence and overall depression scores reported in females in previous studies (48). These findings are consistent with those of Mullarkey et al. (3).

Although core depressive symptoms such as sadness were consistent across genders, feeling bothered and crying showed significant gender differences in edge strength and expected influence, occupying distinct central positions in the male and female networks. These differences may affect how depressive symptoms manifest and spread, highlighting the need to explore gender-specific mechanisms and intervention strategies.

In the ASLEC–CES-D network, the most central symptoms for females were academic stress, illness of a friend, and sadness, whereas for males they were illness, lack of pleasure, and sadness. Regarding bridge symptoms, females were characterized by changes in daily rhythm, school pressure, and frustration, while males exhibited a similar pattern, with school pressure, changes in daily rhythm, and frustration emerging as key bridges. Although some overlap exists, these patterns reveal gender-specific pathways through which negative life events may influence depressive symptoms.

Emerging evidence suggests that hormonal fluctuations during adolescence may heighten emotional sensitivity in females, leading to stronger symptom connectivity and co-activation (49). Moreover, adolescent females are more likely to adopt ruminative coping styles (50), which can amplify negative emotional cascades when facing stressors such as academic challenges, friendship disruptions, or family-related losses (51, 52). These processes may account for the denser interconnections observed among females between negative life events and depressive symptoms.

These mechanisms carry important clinical implications. For females, interventions should prioritize reducing rumination, alleviating academic stress, and strengthening social support networks. In contrast, for males, targeting anhedonia and somatic complaints may be more effective (53). Future studies should employ longitudinal designs to examine the developmental trajectories of gender-specific symptom networks and clarify how these structures evolve throughout adolescence. Furthermore, integrating network approaches with neuroimaging and psychophysiological measures in multimodal research may yield deeper insights into the biological and cognitive mechanisms underlying gender differences in depression.

In summary, this study not only reveals gender-specific differences in the depressive symptom networks of adolescents but also highlights the central and bridge roles of specific symptoms in each gender. These findings provide important theoretical implications for developing more targeted and gender-sensitive psychological interventions.

### 4.2 Limitations

First, the data were obtained through self-report measures, which may be affected by social desirability bias as well as denial or defense mechanisms. Second, this study adopted a cross-sectional design, which limits the ability to infer causal relationships. Future research should employ longitudinal designs to examine the developmental trajectories of depressive symptoms among adolescents, and clinical trials to evaluate whether interventions targeting core depressive symptoms yield superior outcomes. Third, compared with Western adolescents, feeling of failure emerged as a culturally specific core symptom of depression among Chinese adolescents. Further studies are needed to investigate the mechanisms linking feeling of failure and depression in this population, providing an empirical basis for culturally tailored intervention strategies.

## 5 Conclusions

Lack of happiness, depressed mood, feelings of failure, and sadness were identified as the most central symptoms within the depression symptom network among adolescents. The core depressive symptoms were largely consistent across genders; however, significant gender differences were observed in network characteristics, including overall structure, global connectivity strength, edge invariance, and centrality invariance. Notably, female adolescents exhibited significantly higher global connectivity strength than males, indicating a more densely interconnected symptom structure. Furthermore, in the integrated depression–negative life event network, disease, academic stress, and being fined emerged as central nodes. Bridge symptoms—such as flunking, broken heart, and academic stress—played key roles in linking negative life events to depressive symptoms, highlighting potential targets for prevention and early intervention efforts.

## Data Availability

The raw data supporting the conclusions of this article will be made available by the authors, without undue reservation.
